# Review on the Application of Machine Learning Algorithms in the Sequence Data Mining of DNA

**DOI:** 10.3389/fbioe.2020.01032

**Published:** 2020-09-04

**Authors:** Aimin Yang, Wei Zhang, Jiahao Wang, Ke Yang, Yang Han, Limin Zhang

**Affiliations:** ^1^College of Science, North China University of Science and Technology, Tangshan, China; ^2^College of Yi Sheng, North China University of Science and Technology, Tangshan, China; ^3^Mathmatics and Computer Department, Hengshui University, Hengshui, China

**Keywords:** DNA sequence, machine learning, data mining, DNA sequence alignment, DNA sequence classification, DNA sequence clustering, DNA pattern mining

## Abstract

Deoxyribonucleic acid (DNA) is a biological macromolecule. Its main function is information storage. At present, the advancement of sequencing technology had caused DNA sequence data to grow at an explosive rate, which has also pushed the study of DNA sequences in the wave of big data. Moreover, machine learning is a powerful technique for analyzing largescale data and learns spontaneously to gain knowledge. It has been widely used in DNA sequence data analysis and obtained a lot of research achievements. Firstly, the review introduces the development process of sequencing technology, expounds on the concept of DNA sequence data structure and sequence similarity. Then we analyze the basic process of data mining, summary several major machine learning algorithms, and put forward the challenges faced by machine learning algorithms in the mining of biological sequence data and possible solutions in the future. Then we review four typical applications of machine learning in DNA sequence data: DNA sequence alignment, DNA sequence classification, DNA sequence clustering, and DNA pattern mining. We analyze their corresponding biological application background and significance, and systematically summarized the development and potential problems in the field of DNA sequence data mining in recent years. Finally, we summarize the content of the review and look into the future of some research directions for the next step.

## Introduction

We live in the era of the genome, advances in science have allowed humans to spy on the mysteries of life. In recent decades, the rapid expansion of biological data is a significant feature of the development of molecular biology, and a massive biological information database has rapidly formed. We must obtain useful knowledge from these huge data, and simultaneously bioinformatics was born. Bioinformatics is an interdisciplinary subject. It comprehensively uses mathematics, life sciences, and computer science to mine biological information in biological data (Chu, 2014), and further guides the relevant researches of biological researchers. Specifically, the first step is to obtain information on the protein-coding region by analyzing the genomic DNA sequence. Then simulating and predicting the spatial structure of the protein. Finally, according to the function of the protein, the researchers make the necessary drug design.

According to statistics, the amount of biological data approximately doubles every 18 months. In 1982, GenBank’s first nucleic acid sequence database had only 606 sequences, containing 680,000 nucleotide bases (Bilofsky et al., 1986). As of February 2013, its database already contains 162 million biological sequence data, containing 150 billion nucleotide bases. How to mine knowledge from these huge data and guide biological research s an important research content of bioinformatics.

For complex biological data, on the one hand, it is necessary to solve the problem of storage and management of massive data, and on the one hand, it is necessary to extract effective information from the data on the premise of ensuring that the data reflects the true meaning of biology. Machine learning is an important method to achieve artificial intelligence. It can handle the automatic learning of machines without explicit programming and has been widely used in the field of bioinformatics (Li et al., 2005; Larranaga et al., 2006).

DNA is a kind of biomacromolecule in organisms. It carries the genetic information of life and guides the development of biological development and the functioning of life functions. At present, machine learning has been widely used in sequence data analysis and has very broad application prospects in improving data processing capabilities and generating valuable biological information. The review focuses on DNA sequence data mining and machine learning. The review briefly introduces the development process of sequencing technology, DNA sequence data structure, and several sequence encoding methods in machine learning. And we clarify that sequence similarity is the basis of DNA sequence data mining. We have comprehensively analyzed the basic process of data mining and summarized the algorithms commonly used in machine learning. Then, we summarized four typical applications of machine learning in DNA sequence data: DNA sequence alignment, classification, clustering, and pattern mining. In summary, we have the following conclusions: distributed sequence alignment and parallel computing may be the research focus of DNA sequence alignment. How to effectively express sequence features and analyze DNA sequence classification is a difficult point in research. The two key points of DNA sequence clustering are how to extract characteristic subsequences in the DNA sequence. DNA sequence pattern mining will generate an explosion of candidate sequence patterns, which will consume a lot of time and space. How to design a suitable search strategy and eliminate redundant sequence patterns will be an important direction for future research.

## Basic Knowledge of DNA

Gene sequencing is one of the most popular technologies in life sciences. At present, HiSeq X Ten is the sequencing platform with the highest sequencing throughput and the lowest cost. The introduction of equipment and its commercialization has greatly promoted the development of the sequencing industry. The rapid progress of sequencing technology and the continuous decline of sequencing costs have made sequencing more and more common.

Sequence similarity is the basis of sequence data mining, and it is a research direction where sequence similarity bioinformatics is very meaningful. Sequence similarity refers to the degree of similarity between sequences. If the similarity between two sequences exceeds 30%, it is considered that they may have homology. The homologous sequences have a common evolutionary ancestor, and their structures and functions may have similarities.

### Development of Sequencing Technology

**FIGURE 1:**
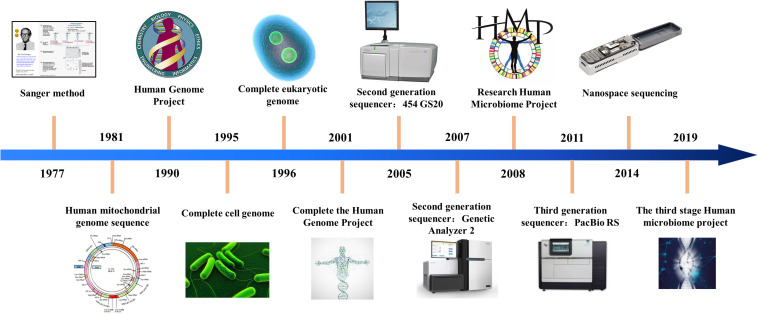
History of sequencing technology.

With the development of biological information technology, sequencing technology has experienced three stages of development. The chain termination method proposed by Sanger and the chain degradation method proposed by Gibert is collectively called the first generation sequencing technology. At present, Sanger sequencing is still widely used in conventional sequencing applications and verification, but the sequencing cost is extremely high and the throughput is low, which seriously affects its truly large-scale application. After more than 40 years of technological development, sequencing technology has achieved considerable progress. The progress of sequencing technology is shown in Figure 1.

After the continuous efforts of researchers, the second generation sequencing technology marked by 454 technology was born in 2005. These sequencing systems can analyze billions of sequencing reactions at the same time. The second-generation sequencing technology is a kind of connected sequencing, which greatly improves the speed of sequencing and greatly reduces the cost of sequencing. At present, the second-generation sequencing technology (Watson, 2014) is the main force in the scientific research market. Due to its low cost, it has been widely used.

In 2011, the third generation sequencing technology represented by Oxford single molecule sequencing technology and PacBio’s SMRT technology was born. Single molecule sequencing is the biggest feature of the third-generation sequencing technology. This technology needs to be continuously adjusted and upgraded for large-scale applications. Sequencing technology is revolutionizing personalized medicine by providing high throughput options with sequence capabilities for clinical diagnosis.

Genomic big data analysis is becoming the next frontier in the field of biomedicine (Roukos, 2010), which integrates data storage, data sharing, data analysis, and data quality control. The sequencing error rate profiles of different sequencing platforms are different, so we need to know which sequencing platform is used to generate the original data, what is their error rate distribution, and whether there are certain biases and limitations. At present, the three major international biological data centers (NCBI, EBI, and DDBJ) have established a series of biological information databases and various data services, which provide strong support for biological data analysis. Biomedical data presents the characteristics of a wide variety, high-dimensional complex internal structure, rich content, relatively scattered data, and difficulty in high-dimensional multi-level cross-sharing.

### Data Structure of DNA Sequence

Biological studies have shown that biological sequences are not random and unordered strings. They consist of a linear arrangement of smaller elements. The DNA sequence is connected by four kinds of deoxyribonucleotides (bases). Base order contributes to the diversity of DNA molecules.

**FIGURE 2:**
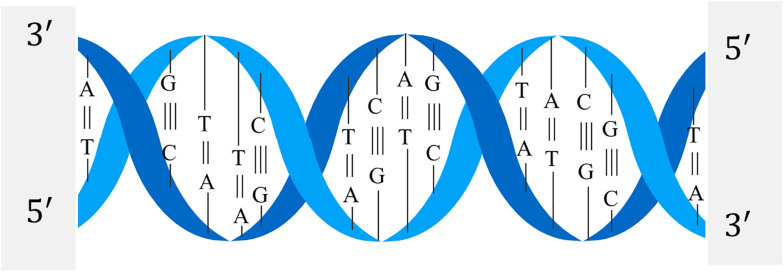
Double helix of DNA.

The structure of the DNA double helix is shown in Figure 2. The nitrogen-containing bases of one strand of the DNA double helix structure will only bond with specific bases of the other strand. It is generally called complementary base pairing, and a base pair is the basic unit of DNA sequence.

DNA sequence data have different characteristics from other data, mainly including:

1. DNA sequence data consists of non-numeric (A, T, C, G) characters;
2. The length of different sequences varies greatly. Some sequences have only a few dozen characters, while others are very long, up to hundreds of megabytes;
3. DNA sequence data contains its specific biological significance;
4. Due to certain errors in the sequencing process and noise in the sequence data, it is necessary to perform corresponding data preprocessing before analyzing the data.

### DNA Sequence Coding

When processing the DNA sequence, it is necessary to convert the string sequence into a numerical value, so as to form a matrix input model training. Generally speaking, there are three methods for sequence encoding: sequential encoding, one-hot encoding, and k-mer encoding (Choong and Lee, 2017). The characteristics of the three DNA encoding methods are shown in Table 1. The performance of sequential encoding is comparable to one-hot encoding, but the training time is significantly reduced. One-hot encoding is widely used in deep learning methods and is very suitable for algorithms such as CNN (convolutional neural networks). In addition, the performance of one-hot encoding is quite consistent in different data sets, but a suitable CNN is required to get good performance. Ordinal codes represented by matrices perform best in some evaluation data sets. The performance of CNN in discovering DNA motifs depends on the proper design of sequence encoding and representation. The good performance of the ordinal coding method shows that there is still room for improvement in the single-point coding method.

**TABLE 1:** Common ways of encoding DNA sequences.

**Encoding method**	**Features**
Sequential encoding	This method encodes each base as a number. For example, change [A,T,G,C] to [0.25, 0.5, 0.75, 1.0], and any other character can be recorded as zero.
One-hot encoding	This method is widely used in deep learning methods. For example, [A,T,G,C] will become [0,0,0,1], [0,0,1,0], [0,1,0,0], [1,0,0,0]. These coded vectors can be connected or turned into a two-dimensional array.
K-mer encoding	First take a longer biological sequence and decompose it into k-length overlapping fragments. For example, if we use a segment of length 6, “ATGCATGCA” will become: “ATGCAT,” “TGCATG,” “GCATGC,” “CATGCA.”

### DNA Sequence Similarity

The main mining modes of machine learning include data characterization and differentiation, data frequent patterns, association and correlation, classification and regression of data predictive analysis, cluster analysis, and outlier analysis. Data mining for DNA sequences is generally carried out from these aspects, and research in these areas is inseparable from similarity analysis between sequences (Pearson, 2013). It can be seen that sequence similarity is the basis of DNA sequence data mining.

Sequence similarity means that there are similar or identical sites between sequences. The sequence similarity can be a quantitative value or a qualitative description. If the degree of similarity between two sequences exceeds 30%, It is considered that the two sequences have a homologous relationship. Therefore, if the two sequences are highly similar, the two sequences are likely to have a common evolutionary ancestor. At the same time, if a sequence similar to the unknown sequence can be found from the sequences with known functions, we can further predict the function (Rogozin et al., 1996) of the unknown sequence.

One of the main problems of DNA sequence similarity research is to search for sequences whose similarity to a specified sequence exceeds a certain threshold. The most commonly used method is to establish a similarity matrix (Henikoff and Henikoff, 1992) and find the best match between sequences in consideration of possible insertions, deletions, and mutations. The study of sequence similarity is divided into global similarity research and local similarity research. The global similarity is the similarity matching of the entire sequence, which is suitable for sequences with a high degree of similarity at the global level. The Needleman-Wunsch algorithm is a typical sequence alignment algorithm (Pearson and Lipman, 1988). However, genes only account for about 2% of the DNA sequence, that is, only a few sequence fragments have a functional role. Although there is no similarity between sequences as a whole, there are similarities in some local areas. Therefore, it is more meaningful to study local similarity than global similarity. Typical local alignment algorithms include the Smith-Waterman algorithm based on dynamic programming algorithm and heuristic database similarity search algorithms FASTA and BLAST (basic local alignment search tool). In a recent study, Delibas and Arslan (2020) proposed a non-aligned sequence similarity analysis method, a new method of DNA sequence similarity analysis using the similarity calculation of texture images, which is a digital image processing method.

Sequence similarity is one of the key processes of DNA sequence analysis in computational biology and bioinformatics. In the study of gene function analysis, protein structure prediction and sequence retrieval, similarity calculations are required. We select the appropriate sequence similarity analysis method and improve it according to actual application requirements and biological background. This is the basis and key of DNA sequence data mining.

## Machine Learning Algorithm

In the past few decades, we have witnessed the revolutionary development of biomedical research and biotechnology and the explosive growth of biomedical data. The problem has changed from the accumulation of biomedical data to how to mine useful knowledge from the data. On the one hand, the rapid development of biotechnology and biological data analysis methods has led to the emergence of a challenging new field: bioinformatics. On the other hand, the continuous development of biological data mining technology has produced a large number of effective and well-scalable algorithms. How to build a bridge between the two fields of machine learning and bioinformatics to successfully analyze biomedical data is worthy of attention and research. In particular, we should analyze how to use data mining for effective biomedical data analysis, and outline some research questions that may stimulate the further development of powerful biological machine learning algorithms.

### Basic Process of Data Mining

**FIGURE 3:**
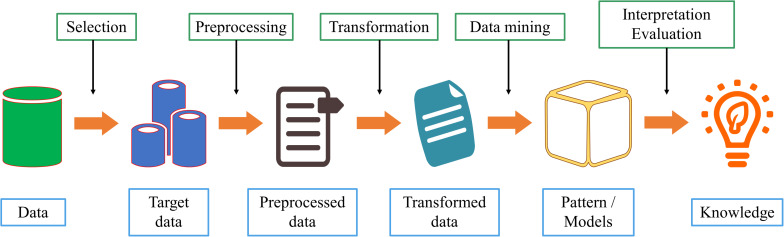
The steps for data mining process.

Data mining is a discipline that combines classic statistical tools with computer science algorithms. This discipline aims to mine knowledge from large amounts of data for scientific, computational, or industrial use. As shown in Figure 3, we comprehensively describe the process of data mining from six aspects.

1. Data cleaning. Because of the increasing amount of heterogeneous data, data sets often have missing data and inconsistent data. Low data quality will have a serious negative impact on the information extraction process. Therefore, deleting incomplete, or inconsistent data is the first step in data mining;
2. Data integration. If the source of the data to be studied is different, it must be aggregated consistently;
3. Data selection. Accurately select relevant data based on the research content;
4. Data conversion. Transform or merge data into a form suitable for mining, and integrate new attributes or functions useful for the data mining process;
5. Data mining. Select the appropriate model according to the problem and make subsequent improvements;
6. Mode evaluation. After acquiring knowledge from the data, select appropriate indicators to evaluate the model.

The main task of the data mining step is to correctly select one or a combination of these steps and find an effective and reliable method to solve the given problem. In recent years, machine learning has been widely used in bioinformatics analysis. Each step of data mining is developed independently of other steps, and each step has a large number of machine learning algorithms.

### Association Rule Mining Algorithm

As one of the most important branches of data mining, association rule mining can identify the associations and frequent patterns of a set of items in a given database. It consists of two sub-problems: (1) Set the minimum support threshold and use the minimum support Find frequent itemsets from the database; (2) Use minimum confidence to find association rules that satisfy specified constraints on frequent itemsets. Association rule mining not only plays an important role in business data analysis but has also been successful in many other fields, such as virtual shopping basket analysis and medical data analysis.

The *Apriori* algorithm is a typical association rule-based mining algorithm, which has applications in sequence pattern mining and protein structure prediction. Many machine learning algorithms in data mining are derived based on *Apriori* (Zhang et al., 2014). The basic method of association rule mining is through the use of Some metrics are used to analyze the strong associations in the database. The most commonly used measurement methods are minimum support and minimum confidence. The *Apriori* algorithm uses a guided method to mine association rules between data items in the database.

### Classification Algorithm

Classification is one of the most studied tasks in machine learning. The principle of classification is based on the predicted attribute to predict the class of the target attribute specified by the user. In genomics, the key issues are genome classification and sequence annotation. In the mining of biological sequences, widely used algorithms include fuzzy sets, neural networks, genetic algorithms, and rough sets. There are also many general classification models, such as naive Bayesian networks, decision trees, neural networks, and rule learning using evolutionary algorithms.

### Clustering Algorithm

The clustering algorithm in machine learning can cluster together sequences with some same characteristics, and explore the effective information of unknown sequences from known functions and structures. Therefore, the clustering of biological sequences is of great significance to the research of bioinformatics. The difference from the classification is that clustering does not implement a set category. Each cluster has its own common characteristics. The purpose of cluster analysis is to divide the data with common characteristics into one category, then use other methods to analyze the data.

In recent years, with the development of artificial intelligence, the clustering algorithm has become a popular research direction in the field of machine learning. To improve the processing capacity of large scale data, domestic and foreign scholars have conducted more in-depth research on clustering algorithms. Several excellent clustering algorithms have emerged: there are mainly clustering algorithms based on granularity, clustering algorithms based on uncertainty, clustering algorithms based on entropy, clustering integration algorithms, etc.

Besides the above-mentioned ones, there are a large number of algorithms. Each algorithm has its characteristics, an algorithm cannot be applied in all situations. Understand the advantages and disadvantages of each algorithm could help us better use and research.

### Challenges and Future Solutions

Machine learning is the core of data mining and the most widely used data processing method. A key advantage of machine learning algorithms is that they can be used to filter large amounts of data to explore patterns that may be overlooked. In the era of big data in biomedical research, machine learning plays a key role in discovering predictable patterns in biological systems. The current application of machine learning in biomedical data mainly has the following problems:

1. Large data sets are the key to machine learning. At present, the magnitude of most biological data sets is still too small to meet the requirements of machine learning algorithms. Although the total amount of biological data is huge and increasing day by day, the collection of data comes from different platforms. Due to the differences in technology and biology itself, it is very difficult to integrate different data sets;
2. Due to the differences in biological data itself, machine learning models trained on one data set may not be well generalized to other data sets. If the new data is significantly different from the training data, the analysis results of the machine learning model are likely to be false;
3. The black-box nature of machine learning models brings new challenges to biological applications. It is usually very difficult to interpret the output of a given model from a biological point of view, which limits the application of the model.

Machine learning presents new opportunities and challenges to the development of life sciences. In response to the above issues, we believe that future research directions should include the following:

1. The first is to collect large and well-annotated data sets;
2. A certain machine learning model cannot apply to all data sets, so any new data set should match the general attributes of the data used to train the model;
3. We urgently need to develop a means to transform the “black box” of machine learning into a biologically meaningful and interpretable “white box.”

There are many opportunities at the intersection of machine learning and biomedical data integration, but there are also huge challenges to overcome. Machine learning itself is far from realizing its potential in the field of biological research, and we still have a long way to go.

## Application of Machine Learning in DNA Sequence Data Mining

Machine learning is an important branch of computer science. On the one hand, machine learning makes it possible to mine useful knowledge from large data sets. On the other hand, many areas are also eager to obtain knowledge from data to guide practice. Machine learning also provides new opportunities and challenges for the development of these areas. The benign interaction brought about by this interdisciplinary integration has undoubtedly promoted the development and prosperity of machine learning.

DNA is a biological macromolecule and the basic unit of biological genetic material. Its main function is the storage of genetic information. The calculation and analysis of DNA sequences had undergone fundamental changes in the 1980s. As the genome sequencing system continues to develop, the study of DNA sequences has gradually shifted from the accumulation of original data onto the interpretation of data. This section summarizes the four applications of machine learning in DNA sequence data: DNA sequence alignment, classification, clustering, and pattern mining, and analyzes and discusses the corresponding biological application background and significance. Finally, we systematically summarize the research in the field of machine learning in recent years.

### DNA Sequence Alignment

Sequence alignment is the comparison of two or more sequences in the order of base arrangement, mainly to compare sequences with unknown functions to sequences with known sequences. And the results of the alignment reflect the similarity between sequences and their biology Features. Sequence alignment analysis is one of the most basic and important issues in bioinformatics. Through sequence alignment analysis, the structure and function of biological sequences can be further predicted. According to the study of biology, the evolution of DNA has the possibility of gene recombination and mutation, and the evolutionary process of DNA has been unable to recover and reproduce. However, evolution can be studied to explore the homology between DNA through sequence alignment analysis.

**FIGURE 4:**
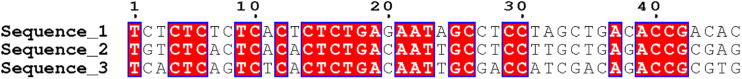
DNA sequence fragment alignment diagram.

Sequence alignment can be divided into double sequence alignment and multi-sequence alignment. Multi-sequence alignment is an extension of double sequence alignment. As the number of sequence alignments increases, the difficulty of alignment is also greater. At present, the research of biological sequence alignment is very mature, and a large number of sequence alignment tools have appeared, such as CLUSTAL, TCOFFEE, and MUSCLE. We selected three DNA sequences of equal length and used CLUSTAL software for sequence comparison. The local visualization of the comparison results is shown in Figure 4. The red area indicates the part of the three sequences that are completely matched. The number of completely matched bases in the figure is 25. The number of bases in the sequence fragment is 46. The sequence similarity reaches 54.35%, and it can be considered that the three sequences have local similarities. Figure 4 is just the simplest comparison situation. In the actual sequence comparison, the situation is much more complicated.

**FIGURE 5:**
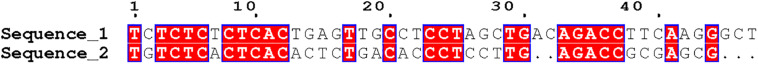
Non-isometric DNA sequence alignment diagram.

At the early stages, research on biological sequence alignment started with dual sequence alignment. Needleman and Wunsch used dynamic programming algorithms for dual sequence alignment based on the similarity of the entire sequence, this is the Needleman-Wunsch algorithm commonly used in sequence alignment, which is also known as global sequence comparison method and optimization matching algorithm. Smith and Waterman (1981) improved the dynamic programming algorithm to make it into a local optimal algorithm, which can search for sequence fragments with the high local similarity between two sequences. The disadvantage of the Smith-Waterman algorithm is that the comparison speed is slow. If you want to search for the maximum matching base number of two DNA sequences, you need to find the longest common substring of the two sequences. First, calculate the score matrix of the double sequence alignment, and then use the dynamic programming algorithm to obtain the matched string. We selected two DNA sequences of non-equal length and used CLUSTAL software for sequence comparison. The local visualization of the comparison results is shown in Figure 5. Because the number of bases in the two DNA sequences is not equal, it is necessary to insert blanks to search for the maximum number of matched bases. The number of bases for a perfect match is 25, and the local similarity is also very high.

Later, BLAST and FASTA have important applications in the query and search of biological sequence databases. With the deepening of research, the swarm intelligence algorithm and its improved algorithm gradually began to be applied to biological sequences alignment, such as the genetic algorithm, ant colony algorithm, etc. Jangam and Chakraborti (2007) proposed a double sequence alignment hybrid algorithm based on a genetic algorithm and ant colony algorithm. The algorithm combines the local feature search capability of the ant colony algorithm and the global feature search capability of the genetic algorithm. The algorithm greatly optimizes the sequence alignment results. For short and medium sequences, the algorithm has high accuracy and better performance than the basic genetic algorithm, but the search efficiency for long sequences is low. To solve the problems of slow convergence and easy local optimization of the ant colony algorithm, Zhao et al. (2008) proposed a sequence comparison method based on an improved ant colony algorithm. By adjusting the initial and final positions of the ants and modifying the pheromone at different times, the algorithm solves the problem that the result falls into a locally optimal solution, but the amount of calculation is large, and it takes a long time to solve.

Multi sequence alignment (MSA) is an extension of double sequence alignment, but when the amount of sequences is large, it will face the problem of excessive data storage space occupation and high calculation complexity. MSA has a key characteristic: Since MSA is an NP-complete problem, MSA relies on approximate alignment heuristic algorithms. These heuristic algorithms depend to a certain extent on specific data attributes. This algorithm was proposed by Hogeweg, and later researchers developed sequence alignment packages based on it, such as CLUSTAL, T-Coffee, CLUSTALW. In recent years, the research and application of iterative algorithms in MSA have become common. Huo and Xiao (2007) proposed a graph-based DNA multi-sequence alignment algorithm: MWPAlign. This algorithm expresses sequence information as a structure graph and converts the sequence alignment problem into the maximum weight path of the graph. The algorithm has a linear time complexity, which significantly reduces the problem of excessive time complexity caused by MSA. However, when the mutation rate between sequences is different, the comparison result is poor, and the algorithm itself loses sequence similarity information in the process of looping. Lee et al. (2008) proposed a multi-sequence alignment genetic algorithm (GA-ACO) with ant colony optimization. GA-ACO algorithm combined with local search. GA-ACO uses ant colony optimization (ACO) to enhance the performance of GA. In the GA-ACO algorithm, GA guarantees the diversity of comparisons, and ACO avoids the result falling into a locally optimal solution. The hybrid genetic algorithm solves the problem of large-scale calculations, but the search speed of the algorithm is relatively slow, and more accurate solutions require more training time.

Naznin et al. (2011) proposed a method of multi-sequence alignment using genetic algorithm vertical decomposition (VDGA). The algorithm uses two mechanisms to generate the initial population: (1) generate a guide with randomly selected sequences Trees; (2) Combine sequences in such trees. VDGA divides the sequence vertically into two or more subsequences, then uses the guide tree method to solve them separately, and finally combines all the subsequences to generate a new multiple sequence alignment. After statistical and experimental analysis, VDGA is an effective method to solve the problem of multiple sequence alignment. The tree model is the most widely used in the field of machine learning, and it is also a model with many variants. The tree model is easy to understand and not easy to overfit, and it consumes fewer resources during training. So tree models are also often used in the biological sequence alignment.

Many studies have focused on heuristic techniques to solve MSA problems, among which stochastic methods are very effective methods. GA is a stochastic method, which can solve this type of optimization problem well. Chowdhury and Garai (2017) summarized the DNA multiple sequence alignment from the perspective of a genetic algorithm. Genetic algorithm has the following advantages in MSA:

1. You can find the optimal solution or the suboptimal solution of the sequence alignment problem in computing time;
2. Regardless of the length of the sequence and the number of sequences, this method is applicable;
3. There is much room for improvement in the optimization of the objective function, and the description of the objective function is crucial for the optimal solution of sequence alignment.

The scale of biological sequence data continues to grow, and sequence alignment is a necessary step for sequence data analysis. Since the research of sequence alignment is very mature, a large number of excellent and open-source sequence alignment tools have appeared. At present, the research of sequence alignment focuses on improving the speed of the alignment. Faced with such a large amount of sequence data, traditional sequence comparison tools can no longer handle it, so highly distributed computers will be required. In recent years, a distributed computing framework called Hadoop can be used for big data processing and storage. It has two main components, MapReduce for programming model and Hadoop Distributed File System (HDFS) for storing data. Using the distributed platform of the MapReduce model, massive sequencing data can be effectively stored and analyzed. Mondal and Khatua (2019) proposed a distributed sequence alignment algorithm: MRaligner. The algorithm is implemented in the Apache Spark framework using MapReduce. Compared with the traditional Smith-Waterman algorithm, the sequence comparison efficiency has been significantly improved. Besides, because the framework is flexible and extensible, increasing the number of processors and good distributed HDFS management will speed up processing.

Evaluation of biological sequence alignment algorithms mainly considers the efficiency of the algorithm and the sensitivity to obtain the best alignment results. The Smith-Waterman algorithm for double-sequence alignment is highly sensitive, but its complexity is high. FASTA and BLAST are a decrease in predicted sensitivity in exchange for an increase in speed. The CLUSTALW algorithm is the most common and effective among multiple sequence alignment algorithms. The main problem in sequence alignment is whether the sensitivity of the alignment and the efficiency of the algorithm have been improved for sequences with large differences.

Next-generation sequencing technology (NGS) has brought us a lot of biological data. Sequence alignment is always an indispensable step in finding the relationship between sequences. For fairly large input sequences, sequence alignment is a difficult task Currently, traditional sequence alignment tools are inefficient in terms of computing time. In the future, in the face of high throughput, biological sequence data, distributed sequence alignment, and parallel computing may be the focus of research in this field.

### DNA Sequence Classification

Classification is an important mining task in machine learning. Its purpose is to learn a classification model from the training sample set to predict the category of unknown new samples. The classification of biological sequences as a special data type is a popular problem in data mining. It is a difficult problem, due to the non-numerical attributes of the biological sequence elements, the sequence relationship between the sequence elements, and the different sequence lengths of different events, etc. Sequence classification is to predict the type of DNA sequence based on the similarity of its structure or function, and then predict the sequence function and the relationship between other sequences, and assist in the identification of genes in DNA molecules.

Levy and Stormo (1997) proposed to use circular graphs (DAWGs) to classify DNA sequences. Müller and Koonin (2003) proposed to use vector space to classify DNA sequences. Ranawana and Palade (2005) proposed a multi-classifier system for identifying *E. coli* promoter sequences in DNA sequences. He Uses four different coding methods to encode the sequence and then uses the coding sequence to train four different neural networks. The classification results of the four individual neural networks were then combined through an aggregation function, which used a variation of the logarithmic opinion pool method. Experiments show that when the same data is provided to the neural network with different encoding methods, it can provide slightly different results that can be provided. At the same time, when the results of more classifiers with the same input data are integrated into a multi-classifier, the results we can obtain are better than the single performance of the neural network. However, the main disadvantage of the neural network design is that it is difficult to obtain the optimal parameters of the neural network. This will involve the deployment of the neural network and the optimization of the encoding method used.

Ma et al. (2001) proposed a DNA sequence classification based on the combination of the expectation-maximization algorithm and a neural network, and applied the algorithm to identify the DNA sequence classification of E. coli promoters. Ma Q uses an improved expectation-maximization algorithm to locate the −35 and −10 binding sites in the E. coli promoter sequence. It is no longer assumed that the lengths of the spacers between the binding sites and between the binding sites and the transcription start site are evenly distributed. Instead, he derives the probability distribution of these lengths. According to the information contained in each E. coli promoter sequence, he selects features and uses orthogonal coding methods to represent these features. Finally, these features are input into the neural network for promoter recognition. This method obtained good performance on different data sets.

Zaki et al. (2010) proposed a variable-order hidden Markov model with the continuous state: VOGUE. VOGUE uses a variable sequence mining method to extract frequent patterns with different lengths and spacings between elements, and then he constructs a variable sequence hidden Markov model. Compared with traditional HMM, VOGUE has higher classification accuracy. However, the frequency statistical characteristics of the sub-sequences in the sequence are not considered, which affects the generalization ability of the model.

In recent years, the convolutional neural network is a widely used deep learning model. Convolutional neural networks can extract abstract features from data. Nguyen et al. (2016) used DNA sequences as text data and proposed a new method for classifying DNA sequences with convolutional neural networks. This method uses a one-stop vector to represent the sequence as the input of the model. So, it retains the information of each nucleotide in the basic position sequence. The model was evaluated in 12 DNA sequence data sets. The results show that the model has improved significantly on all these data sets. The continuous development of deep learning has also opened up new ideas for DNA sequence mining.

The machine learning method used for supervised learning classification tasks depends on feature extraction. Bosco and Di Gangi (2016) proposed two different deep learning models. He used the model for classification tasks on five datasets. It turns out that neural deep learning framework or deep learning models can automatically extract useful features from input patterns.

A key problem in genomics is the classification and annotation of sequences. In recent years, a variety of machine learning techniques have been used to complete this task. In any case, the main difficulty behind the problem is still the feature selection process. The sequence has no clear features And the general representation method easily introduces high-dimensional problems. How to effectively represent sequence features and analyze high dimensional data is the difficulty of research.

### DNA Sequence Clustering

Cluster analysis is one of the most commonly used methods of machine learning. It is different from the classification that we don’t know specific categories in advance. Cluster analysis is unsupervised learning of data patterns. DNA sequence clustering is based on sequence similarity analysis. Cluster analysis clusters DNA sequences with similar characteristics into a cluster and then analyzes biological sequence functions. How to determine whether there is a similarity between sequences is the key to DNA sequence clustering. At present, a lot of research in DNA sequence clustering is based on the local characteristics of DNA for clustering, and the clustering results of DNA sequences are affected by many factors Impact. If a clustering algorithm that considers the global characteristics of DNA sequences can be designed, the accuracy of clustering will be greatly improved, and it is of great significance for the further analysis of DNA sequence clusters.

Early foreign scholars Krause et al. (2000) proposed the SYSTERS algorithm, Enright et al. (2002) proposed the GENERAGE algorithm, the basic idea of the two is to calculate the similarity between sequences, and then use a hierarchical clustering algorithm to complete sequence clustering. Gerhardt et al. (2006) proposed a DNA sequence clustering tool based on the concept of graph theory. This method studies the path topology of the biological genome through a triplet network. In this network, the triplets in the DNA sequence are vertices. If two vertices appear side by side on the genome, they are connected. Then the cluster topology is measured to characterize this network topology. Finally, he aims at two main deviations: guanine-cytosine (GC) content and periodicity of DNA sequence base pairs, he constructed some test data of DNA sequences and studied the clustering method based on the constructed random network. The conclusion proves that the clustering coefficient has its research value. Based on the new distance measure DMk, Wei D proposed a new unaligned DNA sequence clustering algorithm mBKM. This method converts the DNA sequence into a feature vector. This method transforms DNA sequences into the feature vectors which contain the occurrence, location, and order relation of k-tuples in the DNA sequence. The mBKM algorithm can effectively classify DNA sequences with similar biological characteristics and discover the relationships between DNA sequences (Wei et al., 2012). However, the method did not consider edge length, and it has not addressed problems with long repeated sequences or long insertions.

Some recent studies have proposed methods for converting DNA data into genomic digital signals. These studies will provide opportunities for existing digital signal processing methods to be used in genomic data. Mendizabal-Ruiz G proposed a method for clustering analysis of DNA sequences based on GSP and K-means clustering. He chose Euclidean distance as the similarity measure to be adopted by the K-means algorithm. This method can be used to evaluate the ability of markers or genes to distinguish organisms at different levels, identify subgroups in a group of organisms, and classify fragments of DNA sequences based on known sequences (Mendizabal-Ruiz et al., 2018). Mendizabal-Ruiz G has demonstrated that it is possible to group DNA sequences based on their frequency components. The future research direction is to determine whether different pyramids occupy the weight of size in sequence clustering.

At present, the two key points of DNA sequence clustering are how to extract the characteristic subsequences in the DNA sequence, and how to design an effective similarity measure from the biological meaning. Based on the above two key points, the design of the DNA sequence clustering algorithm will get a more practical application of clustering results.

### DNA Sequence Pattern Mining

During DNA evolution, its sequence patterns are well conserved, which is of great significance for biological research. The DNA sequence pattern is usually a sequence fragment in the DNA sequence that has a specific function. In the process of DNA evolution, the more conserved regions in most sequences will form specific sequence patterns, and the structure and function of these sequences play an important role. Therefore, identifying these patterns is an important research content of DNA sequence data analysis. This helps to predict DNA sequence function and explain the evolutionary relationship between sequences. The purpose of DNA sequence pattern mining is to find such sequence patterns from DNA sequences and to identify genes and their functions.

Since, Srikant and Agrawal (1996) defined rearranged sequence pattern mining in 1995, related research has become an important field of machine learning. It has attracted the attention of researchers. There are many types of replacement patterns, including interchange item sets, repetitive subsequences, and replacement substructures. DNA sequence pattern mining is to search for replacement subsequences in a sequence.

**FIGURE 6:**
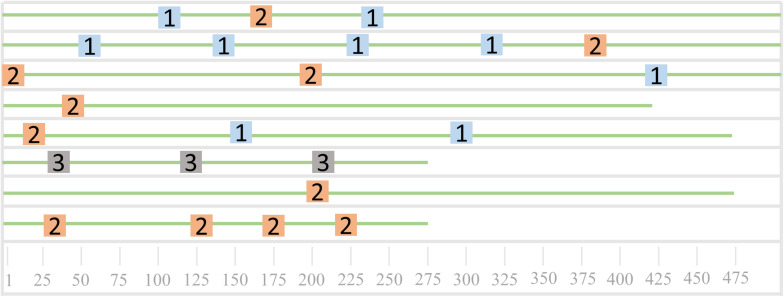
Sequence pattern diagram.

As shown in Figure 6, it is a schematic diagram of the sequence mode. The eight green lines in Figure 6 represent eight sequences, and the three different colored squares represent the three patterns of the sequence. Sequences 3, 5, 6, and 7 all contain the pattern one, sequences 1, 2, 4, 5, 6, and 8 all contain pattern 2, and sequence 3 contains the only pattern three. We can find that both sequences 4 and sequence 8 contain two patterns, which can be used to further analyze the common nature of the two sequences.

The *Apriori* algorithm is commonly used to mine data association rules. It is used to find data sets that frequently appear in data values. Finding out the patterns of these sets helps us make some decisions. Srikant proposed a GSP (generalized sequential patterns mining) algorithm based on the *Apriori* algorithm. The GSP algorithm introduces time and conceptual level constraints and uses a bottom-up breadth-first strategy to mine all frequent patterns (Srikant and Agrawal, 1996). However, when the scale of the sequence database is large, a large number of candidate patterns are generated, and the sequence database needs to be scanned frequently, which leads to the overall efficiency of the algorithm. Therefore, the *Apriori* algorithm is rarely studied alone, but the *Apriori* idea is often used in combination with other algorithms, which will also produce good research results.

At present, there are two main types of calculation methods found in the study of biological sequence patterns: (1) One type uses a heuristic search strategy. This type of algorithm is usually an iterative process. The optimal solution is obtained through repeated iterations. The advantage of the solution is that the calculation complexity is reduced. This kind of method is suitable for the study of subdivided DNA sequences. The disadvantage is that its solution may fall into the local optimum; (2) Another type of algorithm uses an exhaustive search strategy to enumerate all possible solutions and evaluate them one by one to find the best solution.

Existing sequential pattern mining algorithms can be roughly divided into two categories: (1) One type of sequential pattern mining algorithm is to search for patterns in the sequence that exceed a certain threshold: mining alternative patterns. They can only mine alternating patterns in a single sequence. (2) However, in the study of biological sequence analysis, we often require simultaneous analysis of sequence patterns in sequence sets, which cannot be achieved by this method. Therefore, another type of sequence pattern mining algorithm is needed. This type of sequence pattern mining algorithm mines repeated sequence patterns in data sets. When we face massive amounts of biological data, such algorithms usually search very slowly.

Zhou et al. (2010) proposed a pattern mining algorithm: mMBioPM. The algorithm solves the problem of redundancy in the mining results by optimizing the hash table structure with pattern division features, and reduces the calculation time and improves the mining efficiency. To overcome the time complexity and memory overhead caused by a large number of projection databases and short patterns generated by the frequent pattern mining algorithm, Chen and Liu (2011) proposed a fast and efficient biological sequence frequent pattern mining algorithm: FBPM. He defined the concept of the main mode and then used the prefix tree algorithm to mine frequent main modes. At the same time, he used a pattern growth method to mine all common frequent patterns in the sequence.

In the biological sequence database composed of DNA sequences, the existing search algorithm is time-consuming and requires multiple scans of the database. To overcome these disadvantages, Junyan and Chenhui (2015) proposed an SPMM algorithm based on the Markov chain. The algorithm calculates the transition probability matrix of DNA sequences in the sequence database and gives the minimum support threshold as a constraint condition for mining sequence patterns. The calculated degree of support of the subsequence is compared with the threshold to finally determine the sequence pattern. The experiment proves that the SPMM algorithm not only obtains a higher mining speed, but also the mining quality of the sequence mode is higher.

Mao (2019) designed a compact data structure called an association matrix. Based on the association matrix structure, he designed an algorithm for effectively mining key fragments in DNA sequences. The correlation matrix is a novel in-memory data structure. Its structure is very compact and can handle ultra-long DNA sequences in limited storage space. By designing a compact memory data structure and a processing mechanism based on short sequences, it provides a novel idea for analyzing DNA sequences. The effective structure of the correlation matrix can help to efficiently mine key fragments from ultra-long DNA sequences.

DNA sequence pattern mining is a necessary means to study the structure and function of DNA sequences. Traditional DNA sequence pattern mining algorithms will result in a large number of redundant sequence patterns. These sequence patterns are usually short, and they have little biological significance, which makes the results of sequence pattern mining inefficient. At the same time, the long sequence always contains a considerable number of sub-sequences, so an explosive number of candidate sequence patterns will be generated, which will generate a lot of time and space consumption. How to design an appropriate search strategy and eliminate redundant sequence patterns will be an important direction for future research.

### Open Issues

DNA sequence analysis provides an opportunity to explore the genetic variation of organisms. The rapid growth of DNA sequence data has continuously expanded the demand for DNA sequence analysis. At present, there are still the following problems in DNA sequence data mining:

1. There are still efficiency challenges when processing large-scale DNA sequence data;
2. For different biological needs, suitable DNA sequence data mining algorithms should be designed according to the corresponding background knowledge and sequence characteristics;
3. How to extract the sequence characteristics of DNA sequences and how to design an effective similarity measure to measure sequence similarity is very important;
4. Due to the “black box” nature of machine learning, the output of machine learning is difficult to give a reasonable explanation from a biological perspective, which limits the application of the model to a certain extent.

## Conclusion

In the past few decades, the rapid development of hardware technology has opened up new possibilities for life scientists to collect data in various application fields, such as omics, biological imaging, medical imaging, etc. At the same time, the advancement of life science technology has brought Huge challenge. Today, how to apply numerous data mining technologies to bioinformatics analysis is a current research hotspot, including data mining architecture, machine learning algorithm development, and new data mining analysis function research suitable for biological information processing. At the same time, the interdisciplinary approach has promoted the development of machine learning. And artificial neural networks, deep learning, and reinforcement learning have made breakthroughs in machine intelligence. Besides, due to the growth of computing power, the acceleration of data storage speed and the reduction of computing costs,scientists in various fields have been able to apply these technologies to biological data. The close integration of machine learning and bioinformatics will result in more and more meaningful mining results, which will play a positive role in the progress of human society.

Based on the above research, we believe that the research of machine learning in DNA sequence analysis has two aspects that deserve attention:

On the one hand, it describes the biological significance of DNA sequences. At present, a large number of algorithms can achieve efficient performance when analyzing DNA sequences, but their mining results are highly sensitive and specific, which will make a large deviation during use. Therefore, how to integrate the biological significance of DNA sequences into the data mining process is a problem worthy of everyone’s attention and research.

On the other hand, with the continuous expansion of data volume, traditional analysis tools are inefficient in terms of computing time, and how to design efficient calculation methods is an important research aspect. The integration of distributed computing and parallel computing will greatly improve mining efficiency.

At the same time, it is very necessary to choose a suitable DNA sequence coding method for a specific task. This can improve the performance of the algorithm and reduce the training time.

In summary, from the aspects of sequencing technology, DNA sequence data structure, and sequence similarity, this review comprehensively introduces the source and characteristics of DNA sequence data; we briefly summarize the machine learning algorithms and propose biological sequence data Challenges faced by machine learning algorithms in mining and possible solutions in the future. Then, we reviewed four typical applications of machine learning in DNA sequence data: DNA sequence alignment, classification, clustering, and pattern mining, analyzed and discussed their corresponding biological application background and significance, and systematically summarized recent years Research on the field of DNA sequence data mining by domestic and foreign scholars. We put forward several key issues in the future research field of DNA sequence data mining and some future research directions and trends. In future research, I believe that the biological field and machine learning will be more closely integrated, and more effective mining results will be obtained.

## Author Contributions

AY proposed the idea of this manuscript. WZ searched the references. JW wrote this manuscript. KY and YH double checked this manuscript and formatted it as per the template of this journal. LZ polished the language. All authors contributed to the article and approved the submitted version.

## Conflict of Interest

The authors declare that the research was conducted in the absence of any commercial or financial relationships that could be construed as a potential conflict of interest.
